# 3D enamel profilometry reveals faster growth but similar stress severity in Neanderthal versus *Homo sapiens* teeth

**DOI:** 10.1038/s41598-020-80148-w

**Published:** 2021-01-12

**Authors:** Kate McGrath, Laura Sophia Limmer, Annabelle-Louise Lockey, Debbie Guatelli-Steinberg, Donald J. Reid, Carsten Witzel, Emmy Bocaege, Shannon C. McFarlin, Sireen El Zaatari

**Affiliations:** 1 CNRS, MCC, PACEA, UMR 5199, Univ. Bordeaux, Bâtiment B8, Allée Geoffroy Saint-Hilaire, 33615 Pessac, France; 2 Department of Anthropology, Center for the Advanced Study of Human Paleobiology, The George Washington University, 800 22nd St. NW, Suite 6000, Washington, DC 20052 USA; 3 Paleoanthropology, Senckenberg Center for Human Evolution and Paleoenvironment, Universität Tübingen, Rümelinstraße 23, 72070 Tübingen, Germany; 4 Department of Anthropology, Department of Evolution, Ecology and Organismal Biology, The Ohio State University, 4034 Smith Laboratory, 174 W 18th Avenue, Columbus, OH 43210 USA; 5 Skeletal Biology Research Centre, School of Anthropology and Conservation, University of Kent, Canterbury, Kent, CT2 7NZ UK; 6 Department of Biology, Universität Hildesheim, Universitätsplatz 1, 31141 Hildesheim, Germany; 7 Human Origins Program, National Museum of Natural History, Smithsonian Institution, 10th Street & Constitution Avenue NW, Washington, DC 20560 USA

**Keywords:** Biological anthropology, Optical imaging

## Abstract

Early life stress disrupts growth and creates horizontal grooves on the tooth surface in humans and other mammals, yet there is no consensus for their quantitative analysis. Linear defects are considered to be nonspecific stress indicators, but evidence suggests that intermittent, severe stressors create deeper defects than chronic, low-level stressors. However, species-specific growth patterns also influence defect morphology, with faster-growing teeth having shallower defects at the population level. Here we describe a method to measure the depth of linear enamel defects and normal growth increments (i.e., perikymata) from high-resolution 3D topographies using confocal profilometry and apply it to a diverse sample of *Homo neanderthalensis* and *H. sapiens* anterior teeth. Debate surrounds whether Neanderthals exhibited modern human-like growth patterns in their teeth and other systems, with some researchers suggesting that they experienced more severe childhood stress. Our results suggest that Neanderthals have shallower features than *H. sapiens* from the Upper Paleolithic, Neolithic, and medieval eras, mirroring the faster growth rates in Neanderthal anterior teeth. However, when defect depth is scaled by perikymata depth to assess their severity, Neolithic humans have less severe defects, while Neanderthals and the other *H. sapiens* groups show evidence of more severe early life growth disruptions.

## Introduction

The incremental nature of dental development allows researchers to precisely assess the tempo and duration of tooth growth. Aspects of dental ontogeny, including molar eruption patterns, are roughly correlated with the pace of successive life history events like weaning, sexual maturation, and longevity in primates^1^, but much more stands to be learned, particularly among closely related taxa^2,3^. An enduring debate surrounds when the modern human-like life history ‘package’ appeared in our lineage, marked by a relatively longer and slower developmental period compared to other apes^4^. While it is now accepted that species such as *Homo erectus* exhibited a shorter developmental window than our own species, active controversy still exists in regards to the growth patterns of our most recently extinct relative, the Neanderthal^5,6^.

**Figure 1:**
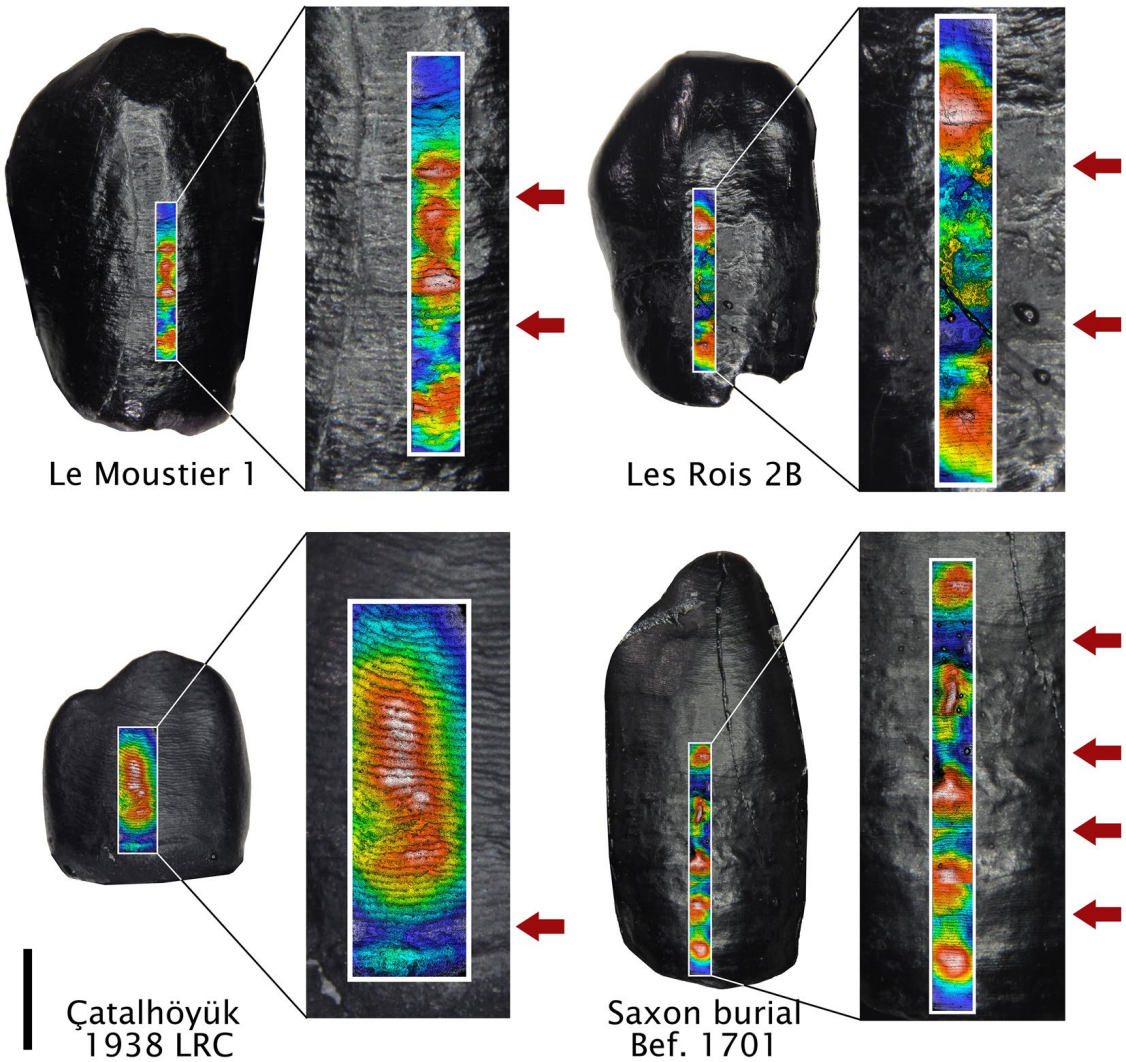
Canine epoxy replicas included in this study. Le Moustier 1 lower right canine (LRC), Les Rois 2B LRC, Çatalhöyük 1938.1 LRC, and Saxon burial at Hildesheim Befund 1701 lower left canine. Digital elevation models generated within SensoSCAN software (S Neox, https://www.sensofar.com/metrology/products/sneox/) are superimposed on the replicas, with blue areas showing surface depressions. Defects are marked with arrows and perikymata are visible as small grooves on the surface. The scale is 3 mm and corresponds to the full replicas.

The most reliable way to glean growth information from teeth is to physically or virtually section them, or image already broken tooth surfaces, thus gaining access to their internal microstructure^7–9^. These methods are inaccessible for most studies due their destructive nature and/or prohibitive cost, and further restricted by their inherent sample size limitations. Instead, many researchers analyze the near-weekly growth increments called perikymata that outcrop on the outer tooth surface (Fig. 1). There have been many advances in the microscopic analysis of perikymata in recent years, allowing for detailed analyses of normal growth patterns as well as growth disruptions caused by early life stress^10–13^.

Stressors like illness, injury, or malnutrition disrupt enamel formation, creating enamel defects on the surface of teeth, the most common of which is linear enamel hypoplasia (LEH)^14^. Hypoplastic defects usually appear as lines or grooves on the enamel surface, following the course of normal perikymata around the tooth crown, but they can also have pits or larger areas of missing enamel^15^. The qualitative analysis of LEH is a mainstay of bioarchaeology and paleobiology, with high LEH prevalence interpreted as a signal of poor living conditions among past populations^14^. A major limitation is that qualitative defect identification makes it difficult to compare results among studies as defects appear on a continuum. Two methods for quantifying LEH defect depth have been proposed^16,17^, but there is yet to be a methodological consensus for data collection and analysis, nor a standard approach for the identification of defects on the basis of their quantitative morphology. Most microscopic analyses of LEH focus on perikymata spacing within defects, but more research is needed to understand and quantify ‘normal’ perikymata variation in different crown regions and tooth types, both within and among species, in order for deviations from normality to be used as an indicator of stress events.

In addition to the replicability of defect identification, measuring LEH defect depth also sheds light on growth and thus life history patterns. At the population level, defect depth has been shown to reflect species-specific enamel growth (i.e., extension) rates in great apes^18^. Faster-growing mountain gorillas have significantly shallower defects, relating to their shallower growth increments below the enamel surface, and mirroring their faster life history patterns overall^19^. Previous work hypothesized that the relationship between enamel growth rates and defect depth exists among hominins as well^20^, with differences in depth, and thus perceptibility via qualitative observation, influencing estimates of LEH prevalence. However, no defect depths are published for *Homo sapiens*, and only one study currently exists on fossil hominins comparing *Australopithecus africanus* and *H. naledi*^21^. Therefore, very little is known about how defect depth might relate to enamel growth and life history patterns in our own species and extinct relatives.

Defect depth also provides information on the severity of growth disruptions themselves, and is best understood by analyzing the cellular activity around the time of the disruption. Evidence from experimental research on sheep incisors suggests that the intensity of stressors influences defect dimensions, with higher doses of growth-disrupting fluoride creating broader and deeper defects^22^. Further evidence from analyses of deer teeth suggest that more severe defects occur in animals exposed to high, intermittent doses of fluoride, particularly in cases where enamel secretion was abruptly halted altogether, with no resumption of cellular activity^23^, forming the equivalent of plane-form hypoplastic defects in primates^24^. In contrast, histological studies of modern human teeth suggest that the classic LEH defect type, also called furrow-form defects, reflect less severe disruptions to late-stage enamel secretion than plane form defects^24^. However, it is often difficult to discern plane- vs. furrow-form defects without histology, the former of which represents a more severe growth disruption from the cellular perspective but of a shorter duration, while the latter often represents a longer-forming but less severe growth disruption^24–26^.

Researchers have long hypothesized that defect depth provides information about the severity of the stressor that disrupted growth in hominoids^16,21,27^, just as location and width of the defects tell us something about their timing and duration, respectively. Evidence from great ape studies supports this, with several wild-captured individuals exhibiting particularly deep defects that might reflect major stress events^17^. One known gorilla exhibits a defect depth of 276 µm (compared to the species median of 41 µm), and according to associated veterinary records, this defect likely formed around the time that she was captured from the wild to live in a zoo^17^. At the population level, mountain gorillas that developed their teeth during a period of intense human encroachment have defects that are almost twice as deep as those that lived under increased protection^17^. Further, flanged orangutans, which exhibit higher levels of the stress hormone cortisol throughout development, have defects that are more than twice as deep as unflanged or developmentally arrested males with either the same or lower cortisol levels^28^. The duration of the disruption is also likely to affect classic furrow-form defect dimensions, as is usually approximated by counting the number of perikymata involved in the defect, with each additional perikyma carving more deeply into the enamel wall^20,21^. At the cellular level, the number of affected enamel-secreting cells, as well as the relative timing of the growth disruption in relation to the life of the cells, affects the depth of the defect at the outer enamel surface^24,25^. Taken together, LEH defect depth is likely to reflect the interaction of multiple factors, including the intensity of the stressor, the duration of the growth disruption, and the developmental timing of the disruption, in addition to interspecific growth variation. However, a growing body of research supports the link between greater stress, as can be defined in terms of intensity and/or duration, and deeper LEH defects, particularly when an effort is made to account for enamel growth variation.

Here, we describe a method to create high-resolution 3D models of the enamel surface using confocal profilometry, thus allowing for the identification and precise measurement of perikymata and LEH defect depths, and apply it to a sample of 17 Neanderthal and 18 *H. sapiens* anterior teeth. Perikymata depth is yet to be quantified in any hominin species despite its potential for providing baseline information about normal growth patterns of the underlying tissue. We compare perikymata and defect depths to published enamel growth information, like enamel extension rates, which vary among tooth types and taxa. Previous work suggests that Neanderthals have enamel extension rates that are an average of 33% higher than European *H. sapiens* in their anterior teeth (28–35%)^29,30^, providing an opportunity to assess defect depth and its relationship to enamel growth patterns in these groups. We scale defect depth by perikymata depth as a means to control for the ‘normal’ interspecific and inter-tooth variation in surface morphology, as this allows for direct comparisons of defect severity, or the relative proportion of enamel that is missing within defects between Neanderthals and *H. sapiens*, as well as among the *H. sapiens* samples. In this way, we are able to test new hypotheses about the severity (i.e., intensity and/or duration) of stress experiences in past populations on the basis of LEH defect depth while accounting for species differences in enamel growth.

Specifically, we aim to:

1. Test whether lines or grooves identified as LEH defects by visual inspection of the enamel surface truly represent localized reductions in enamel thickness by comparing their depth to perikymata depth, and assess the relationship between the two variables;
2. Test whether perikymata and defect depths mirror established growth differences between tooth types and taxa, i.e., whether faster-growing, thinner-enameled incisors have shallower defects than canines, and whether the anterior teeth of Neanderthals have shallower depths compared to Upper Paleolithic, Neolithic, and medieval *H. sapiens*;
3. Use ratios of defect to perikymata depth (i.e., LEH severity ratios) to compare relative stress severity between taxa and among *H. sapiens* samples. We compare the magnitude of the reductions in enamel thickness (i.e., LEH defect depth) while controlling for species-specific variation in perikymata depth, allowing for inferences about the intensity and/or duration of stress episodes to be made.

## Results

We compared defect depths to perikymata depths to ensure that defects identified by eye represent LEH, or localized reductions in enamel thickness, and indeed there is no overlap in their distributions within teeth (N = 280 perikymata and 71 defects) (Table 1; Table S1). There is a significant positive correlation between perikymata and defect depths in the combined sample (R^2^ = 0.39; *p* < 0.001), but a large proportion of variation in defect depth remains unexplained by perikymata depth (Fig. S1). Within individual dentitions, incisors have shallower perikymata (F(1,265) = 143.7, *p* < 0.001) and defect depths (F(1,52) = 21.5, *p* < 0.001) compared to canines, including instances of matched defects across the same dentitions (Table S2).

**Table 1:** Perikymata and LEH defect depths by taxon.

Taxon	Tooth type	N perikymata (pk)	Median and range pk depth (µm)	N defects	Median and range defect depth (µm)	LEH severity ratio (defect/pk depth)
*H. neanderthalensis* (Sites: Le Moustier, La Chaise, La Chaise Suard, Biache-Saint-Vaast, Kulna, Monsempron, Rochelot)	LC LI1 LI2 UC UI1 UI2	10 10 10 50 20 30	1.57 (1.17–3.09) 0.83 (0.71–1.18) 0.84 (0.46–1.78) 1.04 (0.42–3.05) 1.28 (0.49–3.05) 0.81 (0.53–1.43)	5 3 2 13 5 8	26.4 (19.6–46.2) 15.2 (9.8–27.5) 19.0 (10.7–27.3) 24.1 (13.3–45.7) 13.4 (9.5–22.7) 17.2 (10.1–31.3)	16.8 (12.5–29.4) 18.3 (11.8–33.1) 22.6 (12.7–32.5) 27.1 (9.9–49.2) 13.4 (8.7–15.6) 20.7 (13.8–35.6)
*H. sapiens* (Sites: Les Rois, Saint Germain-la-Rivière, Çatalhöyük, Saxon burials at Hildesheim)	LC LI1 LI2 UC UI1 UI2	40 30 30 20 20 10	4.32 (1.28–11.29) 2.42 (0.96–7.95) 2.52 (0.83–8.17) 4.18 (1.68–8.47) 1.79 (1.14–3.09) 2.04 (1.55–3.42)	11 8 6 3 6 1	47.5 (32.8–101.7) 38.4 (20.6–48.4) 36.7 (20.9–50.5) 41.7 (32.7–79.1) 28.4 (18.7–52.4) 29.6	16.1 (5.7–40.0) 19.7 (4.2–46.5) 19.6 (5.6–52.3) 7.8 (6.1–28.3) 16.3 (9.4–30.5) 14.5

**Figure 2:**
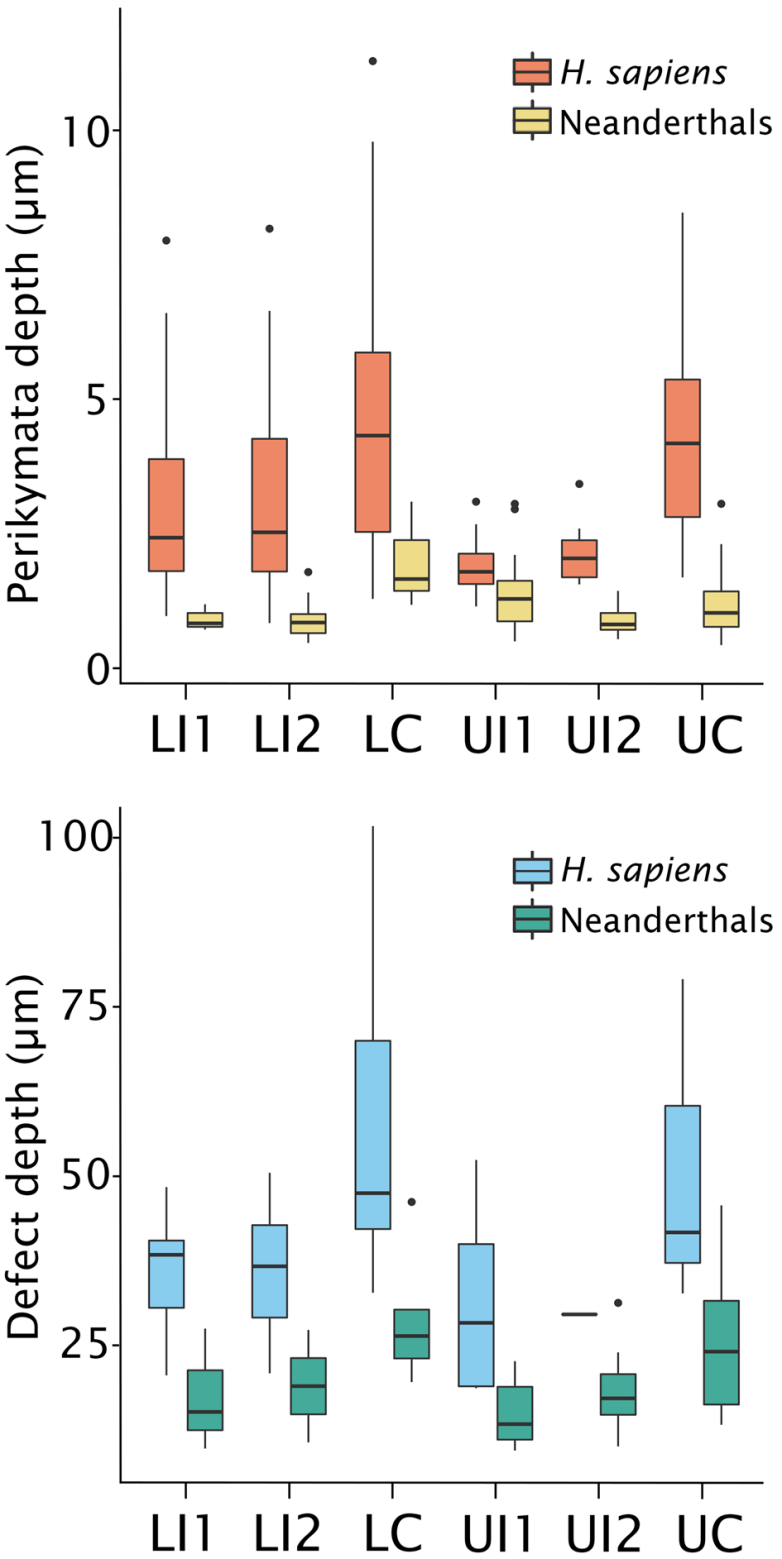
Perikymata and linear hypoplastic defect depths by tooth type. Neanderthals have significantly shallower perikymata (*n* = 280; 150 *H. sapiens* and 130 Neanderthal) and defects (*n* = 71; 35 *H. sapiens* and 36 Neanderthal) than *H. sapiens*. Inter- and intra-individual comparisons suggest that faster-growing and thinner-enameled teeth (i.e., Neanderthals compared to *H. sapiens*; incisors compared to canines) have shallower perikymata and defects at the population level. LLI1/ULI1 = lower/upper central incisor; LLI2/ULI2 = lower/upper lateral incisor; LC = lower/upper canine. Figure generated in RStudio (version 1.3.959)^50^.

The Neanderthal sample has significantly shallower perikymata than the *H. sapiens* sample (F(1,12) = 27.8, *p* < 0.001) (Fig. 2). The *H. sapiens* sample has perikymata that are an average of 2.33 times (range 1.69–3.65) deeper than the Neanderthal sample in incisors, and 3.24 times (range 3.05–3.65) deeper in canines. Perikymata depths range from shallowest in the La Chaise Neanderthal upper canines and incisors to the deepest in the Çatalhöyük lower canines and incisors.

**Figure 3:**
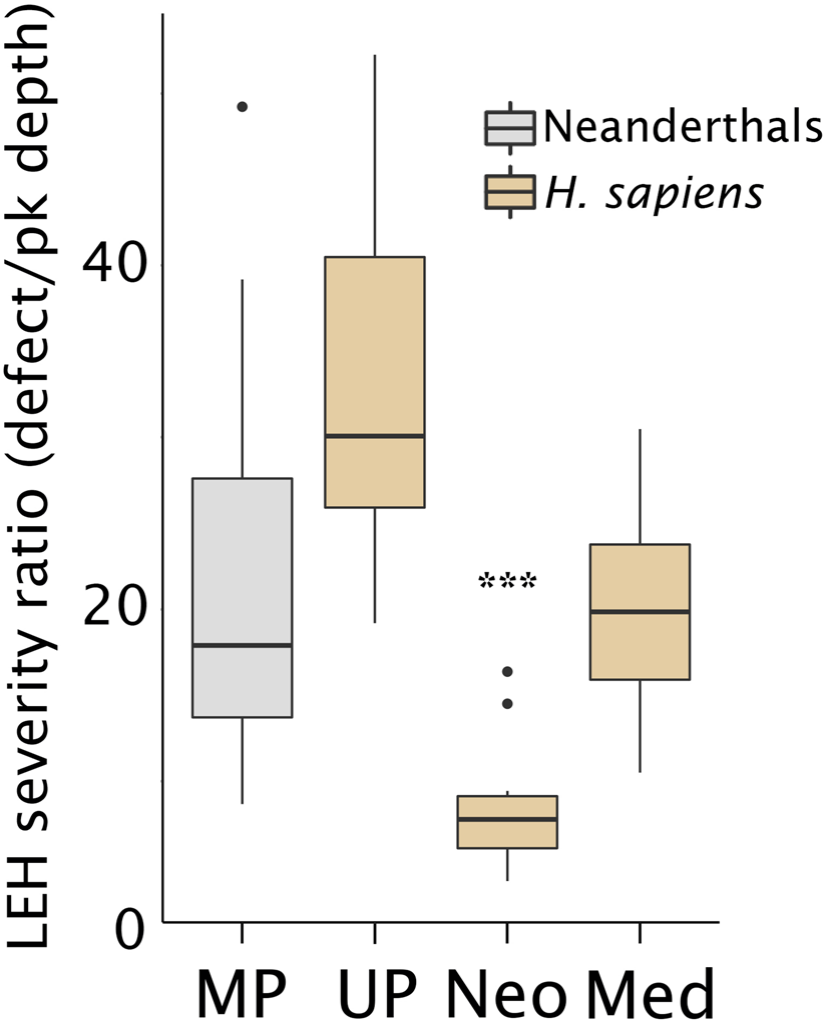
Severity ratios by sample. Ratios are calculated by dividing defect depths by perikymata depths from the same teeth (N = 59), or median perikymata depth for the species and tooth type where perikymata were not well-preserved enough to reliably measure (N = 12). The Neolithic sample from Çatalhöyük (Neo) has significantly lower severity ratios compared to the Medieval (Med), Upper Paleolithic (UP), and Middle Paleolithic (MP) samples, as noted with asterisks. Figure generated in RStudio (version 1.3.959)^50^.

The Neanderthal sample also has significantly shallower LEH defects than *H. sapiens* (F(1,16) = 30.0, *p* < 0.001) (Fig. 2). Defect depths follow a similar pattern with La Chaise Neanderthal upper incisors having the shallowest defects and *H. sapiens* lower canines from the Saxon burials at Hildesheim and Çatalhöyük having the deepest defects. However, there are no significant differences in LEH severity ratios between Neanderthals and *H. sapiens*, as calculated by dividing each defect by the median perikymata depth for each tooth (F(1,16) = 0.30, *p* = 0.598). When further subdivided by sample, the Neolithic Çatalhöyük specimens exhibit significantly lower severity ratios compared to the other *H. sapiens* samples from the medieval and Upper Paleolithic periods, and compared to the Neanderthals (F(3,14) = 15.7, *p* < 0.001; posthoc comparisons—Neolithic vs. medieval *p* = 0.004, Neolithic vs. Upper Paleolithic and Neanderthals *p* < 0.001) (Table 1, Fig. 3).

To test the effect of leveling algorithms that have been used in other studies to remove the effect of curvature^15,20^, and ultimately move toward a methodological consensus for measuring defect depth, plane and sphere-type form removal algorithms (within SensoSCAN) were applied to two digital elevation models (DEMs) from Le Moustier canines (Fig. S2). Leveled values underestimate the depth of defects, being on average 24.3% shallower than raw depths (range = 16.1–27.9%; N = 4), and with a mean absolute difference of 9.9 µm (range = 6.9–11.1 µm; N = 4). When the same algorithms are applied to perikymata, they have the opposite effect, making perikymata appear artificially deeper. The mean difference in perikymata depth is 36.1% (range = 14.3–64.2%; N = 4), with a mean absolute difference of 1.0 µm (range = 0.40–1.63 µm; N = 4).

## Discussion

A long-standing debate surrounds the appearance of modern human-like development and life history. Evidence from studies of brain, skeletal, and dental growth is equivocal, with some researchers arguing that Neanderthals exhibited modern human-like growth patterns, while others suggest that they had faster growth rates, particularly during early development^5,6,29–31^. Our data support the hypothesis that Neanderthals had faster-growing anterior teeth^29^, as evidenced by their shallower perikymata and LEH defects. The extent to which faster anterior tooth growth rates relate to other aspects of life history is yet to be fully understood, but recent evidence suggests that the tempo and mode of brain ontogeny, and possibly skeletal growth, differs between the two species^6^. Among extant apes, faster-growing species like mountain gorillas have shallower LEH defects, faster enamel growth (extension) rates, accelerated brain and somatic growth, and faster life history schedules^17–19,32,33^. However, more comparative data from extant and fossil hominoids are needed in order to further assess the relationships among these variables in hominins.

The application of novel methods such as ours unlocks new information about growth and developmental stress in past populations, improving our understanding of dental development and life history evolution. We provide a way to reliably identify LEH defects on the basis of their being deeper than nearby ‘normal’ growth increments, facilitating cross-study comparisons. When analyzing samples with known differences in their enamel growth patterns, our results show that it is important to take that variation into account when interpreting defect depths. There is a positive relationship between perikymata and defect depth, with deeper defects occurring in teeth with deeper perikymata (Fig. S1). Defect and perikymata depths also follow the predicted pattern based on documented differences in enamel growth rates between different tooth positions, including when comparing matched defects across the same dentitions (Table S2). Shallower features occur in teeth with faster average enamel extension rates and thinner enamel^18^, such as in incisors vs. canines^29,34^. This inter-tooth difference is understood to be a consequence of growth-related variation in underlying enamel geometry, namely the angles that striae of Retzius make as they meet the outer enamel surface^18^. Faster enamel extension rates are also associated with less tightly packed perikymata on the tooth surface^35^, as exhibited by Neanderthals compared to modern humans, particularly in the cervical crown^36^. Neanderthals also have lower periodicities, or the number of days represented by striae of Retzius, providing further evidence of faster growth where perikymata are more widely spaced compared to *H. sapiens*^29^. The Neanderthal sample in this study has two to three times shallower perikymata in their incisors and canines compared to the *H. sapiens* sample, following the same pattern (but at a higher magnitude) as the enamel extension rate differences documented in previous studies^29,30^.

When compared with published defect depths from nonhuman great apes and hominins, the *H. sapiens* sample has the highest median depths (47.5 µm in mandibular canines), perhaps as a result of their slower average extension rates, larger striae of Retzius angles, and thicker enamel compared to apes^18^. The Neanderthal specimens, in contrast, have a median defect depth (30.3 µm) that is more similar to earlier hominins *Australopithecus africanus* (26.0 µm) and *H. naledi* (26.9 µm) as reported by Skinner^21^, as well as nonhuman apes (23.6 µm in mountain gorillas). However, the *A. africanus* and *H. naledi* depths^21^ were collected from leveled DEMs and therefore likely underestimate the true depth, based on our comparisons of leveled vs. raw data (Fig. S2). In terms of differences among the *H. sapiens* samples, relatively little is known about enamel growth variation within our species. A histological analysis of contemporary European and South Africans^37^, and the variation in the timing of dental eruption^38^, suggest that considerable variation can be expected.

If enamel growth variation explains only a moderate proportion of population-level defect depth, we hypothesize that severity—the intensity and/or duration of the stressor—helps to explain the remaining variation^21,39^. We propose that using a ratio of defect to perikymata depth provides a way to tease apart the influence of growth variation (whether tooth- or species-specific) vs. stress severity in defect formation. The quantity of missing enamel tissue within each defect is measured in relation to the local perikymata depth, allowing for comparisons of LEH severity ratios among samples with differing growth patterns. The interplay of severity and duration can further shape the appearance of enamel defects, particularly those of the classic furrow-form type. Guatelli-Steinberg et al.^39^ found that Neanderthal defects represent shorter growth disruptions compared to those in Inuit foragers, and defects of a shorter duration are likely to be shallower than those of a longer duration^21^. Our method is in line with the existing theoretical framework surrounding LEH defect formation in which the intensity of the stressor (i.e., the number of affected ameloblasts), the duration of the stress episode, and the timing of the stressor in relation to cellular developmental stage influence defect depth^15,21,23–26^.

In addition to the life history debate, researchers have long supposed that Neanderthals experienced more severe early life stress compared to Upper Paleolithic humans in Europe^40^. Ogilvie et al.^40^ analyzed a large sample of 669 Neanderthal teeth and suggested that they had high LEH prevalence compared to recent human populations, which they interpreted as a sign of lower foraging effectiveness and higher food stress from the time of weaning through adolescence. Our results show that while Neanderthals have defects that are absolutely shallow, their LEH severity ratios are not significantly different from those in the *H. sapiens* sample as a whole.

When the *H. sapiens* sample is divided by time period, the Neolithic specimens from Çatalhöyük stand out as having significantly less severe LEH (i.e., lower severity ratios) compared to Upper Paleolithic and medieval *H. sapiens* as well as Neanderthals (Table 1, Fig. 3). Çatalhöyük, a well-documented early farming settlement in south-central Anatolia (Turkey; 7100–5150 cal BCE), shows little evidence of early life stress beyond the mere presence of LEH defects, which are mostly short in duration^41^. Skeletal evidence suggests that the ontogenetic patterns at Çatalhöyük match those of well-nourished contemporary populations^41^. The Çatalhöyük specimens included in this study have some of the deepest defects in the sample, but they also have the deepest perikymata, leading Çatalhöyük to have significantly lower LEH severity ratios than the other groups. We hypothesize that deeper perikymata are at least partially explained by slower enamel growth rates, which might also explain their deeper defects in the absence of more severe stress. Expanded analyses incorporating dental histology could test whether enamel growth rates are indeed slower in Neolithic vs. foraging or more recent agricultural populations, fitting with bioarchaeological evidence of reduced energetic investment in maintenance and growth during the transition to agriculture, coinciding with dramatic reductions in stature in many parts of the world^42^.

The reduction in LEH severity ratios is not merely a consequence of adaptive changes through time as the medieval Saxon burials sample has higher ratios than the Neolithic sample. The Saxon burials are derived from a time period of active warfare and increasing population density (c. 700–1030 CE) in modern day Hildesheim, Germany. The Upper Paleolithic individuals have LEH severity ratios that are the highest in the study, but they are not significantly different from the Neanderthals nor the medieval Saxons. While they relied on a lot of the same food resources as Neanderthals, evidence suggests that Upper Paleolithic humans had a more varied and flexible diet, which could have led to lower mortality and higher fertility^43^. Snodgrass and Leonard^43^ hypothesized that Neanderthals would have experienced seasonal periods of “intense energy stress,” and indeed, Smith et al.^44^ found a pronounced week-long internal growth disruption in a Neanderthal tooth that is consistent with illness and associated weight loss during the coldest part of winter. However, little concrete evidence currently exists to help explain the severe LEH in Upper Paleolithic humans.

We suggest that defect depth is the most biologically appropriate signal to measure when attempting to identify and characterize LEH morphology as defects are defined as reductions in enamel thickness. The difference in scale between defect depth vs. changes in perikymata spacing associated with defects makes the former easier to identify, characterize, and verify through repeated and independent measurements. In contrast to spacing-only analyses, which require near-perfect surface preservation, defect depth can be analyzed in any samples with preserved enamel (i.e., present and not covered in calculus or plant matter). Minor variation in perikymata spacing, or accentuated perikymata, could reflect minor growth disruptions, corresponding to the more numerous accentuated lines often visible in thin section. If so, detailed perikymata analyses using the method described here provide a way to quantify those defects that fall in the grey area between clear growth disruptions and ‘normal’ growth increments. Additionally, a better understanding of perikymata packing patterns in both 2- and 3D would contribute to species attributions and assessments of hybridization, as in the case of Les Rois mandible B (included in this study), which displays a mixed morphology of Neanderthal and *H. sapiens*-like traits^45^. By analyzing changes in defect and perikymata morphology through time, it might be possible to determine at what point ‘contemporary’ developmental patterns appeared, if such unique patterns exist. Our preliminary data suggest that recent *H. sapiens* have evolved highly variable perikymata morphology with a wide range of perikymata depths, mirroring the high variation found in other developmental variables like dental eruption times^38^.

At the level of the individual defect, there is evidence to suggest that outliers, or particularly deep defects, reflect more severe stress episodes, i.e., intense in terms of the cellular reaction, physiological stressor, and/or longer-lasting in the case of furrow-form defects^17,21–23^. Only once more data are gathered from extant samples with associated records will models be able to accommodate differential stress experiences as an explanatory variable, and even then, enamel defects will continue to reflect nonspecific stressors in terms of etiology. However, by analyzing defect depth in relation to perikymata depth, information about the magnitude of growth disruptions among populations, as operationalized using the LEH severity ratio, can be gleaned in the absence of associated data. Further, if the relationship between defect depth and enamel extension rates continues to be supported in more taxa, population-level perikymata and defect depth may be used to model aspects of enamel growth in samples that cannot be sectioned or virtually imaged via synchrotron or other methods. Indeed, defects appear on a continuum, and there exists an active debate about to what extent LEH defects reflect ‘normal’ growth processes as opposed to pathologies^46^. This method will ultimately contribute to discussions around interindividual susceptibility to defect formation, and provide more information about the early life stress experiences of individuals. LEH analyses are a standard part of bioarchaeological and paleoanthropological analyses, and it is critical that they strive to take morphological and growth variation into consideration and form interpretations accordingly.

## Materials and methods

### Sample

**Figure 4:**
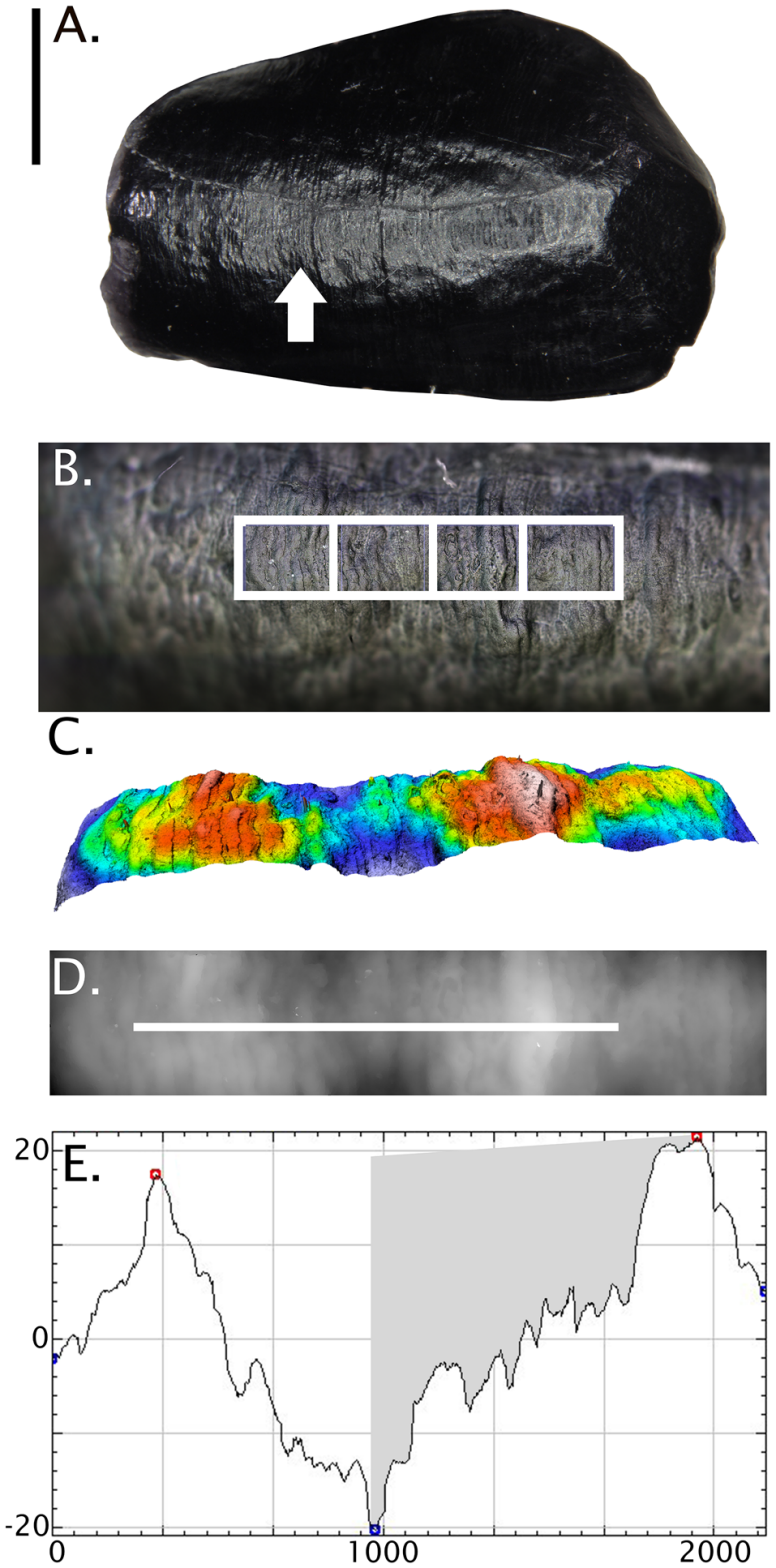
A step-by-step guide to the method. (**A**) Le Moustier 1 LRC epoxy replica with the cusp toward the right of the image (scale = 3 mm). An LEH defect is marked by an arrow. (**B**) An overview image within the SensoSCAN software (S Neox, https://www.sensofar.com/metrology/products/sneox/) with a bounding box marking the crown region to be scanned. (**C**) The resulting digital elevation model (DEM), showing the defect as a reduction in enamel thickness. (**D**) The DEM is read into ImageJ software^47,48^ where a transect is drawn from one occlusal shoulder to the other to extract 2D coordinates for analysis. (**E**) Once plotted, the FindPeaks plug-in^49^ is used to measure maximum defect depth from the occlusal side of the defect (in µm).

**Figure 5:**
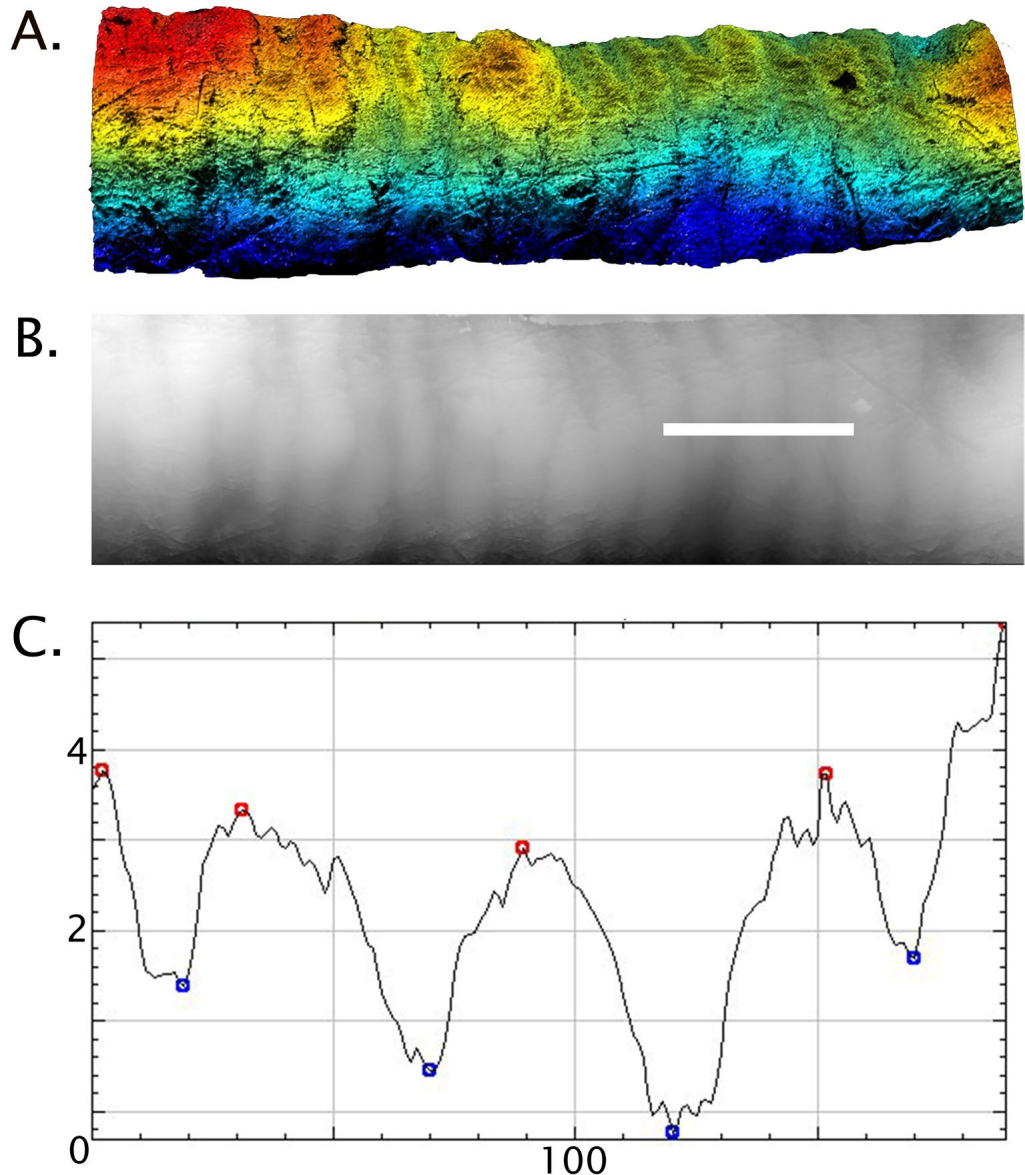
Perikymata measurements in Les Rois 2B lower right canine. (**A**) Digital elevation model (DEM) of midcrown perikymata. (**B**) The same DEM loaded into ImageJ software^47^ using XYZ2DEM plug-in^48^, with a transect drawn across a level region of normal (i.e., without clear defects) perikymata. (**C**) Extracted 2D profile using the FindPeaks plug-in^49^, allowing for the measurement of maximum perikymata depth in four separate furrows (in µm).

The sample includes 35 high resolution epoxy replicas created from permanent anterior teeth (mandibular and maxillary incisors and canines) from *Homo neanderthalensis* (N = 17 teeth; sites: Le Moustier, La Chaise, La Chaise Suard, Biache-Saint-Vaast, Kulna, Monsempron, and Rochelot) and *Homo sapiens* (N = 18 teeth; sites: Les Rois, Saint Germain-la-Rivière, Çatalhöyük, Saxon burials at Hildesheim) (Table S1). Anterior teeth were selected because relatively more of their crowns are made up of imbricational striae, where perikymata and defects are visible on the surface. Specimens were selected for analysis based on their surface preservation, i.e., whether they had visible perikymata and little to moderate wear, calculus, and other debris obscuring the outer enamel surface. Only the best preserved antimere, right or left, was selected for analysis to avoid repeated measurements of the same defects^10,12^. Perikymata and LEH defects were measured within the midcrown defined here as the middle 3/5ths of crown height, avoiding the cuspal and cervical regions most affected by wear and calculus, and reducing the potential influence of changes in underlying geometry on surface feature depth^15^. Defect depth was measured as the maximum difference between the occlusal shoulder and the deepest point within the groove (Fig. 4). Perikymata depth was measured as the maximum difference between the level of a perikyma groove and a perikyma ridge (Fig. 5).

Impressions were collected from original teeth using Coltène’s President Jet Regular and/or Light Body dental impression material (Coltène-Whaledent). High-resolution positive replicas were made using Loctite Hysol E-60NC and/or EPO-TEK 301 epoxy. No coatings or treatments were applied to the replicas for imaging. At least two teeth with partially overlapping developmental periods were analyzed per specimen, except in the case of isolated teeth, to confirm systemic defect manifestation^46^.

### Equipment and settings

Individual defects were first identified via surface visualization (by eye and/or a hand lens) and then imaged using the Sensofar S Neox confocal profilometer. The replica was positioned on the microscope stage using a tilting stage that eases orientation of the defect perpendicular to the light source (Fig. 4A). Once positioned, a quick scan was performed to assess whether the features of interest (i.e., the occlusal shoulders of the target defect or the region with retained perikymata) were level (Fig. 4B). The Z-plane step size for each scan was set to 1 µm with a Z-range encompassing the full height of the defect and the occlusal shoulders, ranging between 75 and 250 µm. The curvature of the crown negatively affects the quality of individual scans and the ability of the researcher to track which defect or perikyma is being measured in later stages of analysis. Therefore, the entire crown, or only crown regions of interest in cases where there is incomplete surface preservation, was imaged in strips of crown height of less than 10 mm each, with 10 percent overlap between image frames (Fig. 4C). The field of view for each scan can be adjusted based on the shape of the tooth. However, a width of at least 500 µm and a length that fully encompasses both the region before and after the defect was used here (e.g., 656 × 3241 µm in Fig. 4B,C).

The ultimate resolution of the Sensofar microscopes provides the appropriate vertical resolution (0.64 µm/pixel; 0.31 µm optical resolution using the 20x lens) to capture the morphology of defects and perikymata as higher resolution images would be too large to analyze on most systems, while lower resolution images would not allow especially perikymata to be reliably measured. The type of light used to probe the surface should be selected based on the material properties of the epoxy replica, but here, white light (as opposed to blue, green, or red) produces the best quality scans. Within the proprietary software SensoSCAN, the confocal fusion algorithm (under expert options) as opposed to continuous provides the highest percentage of measured points (98–99.9%). We provide imaging settings for the Sensofar S Neox profilometer, but other noncontact profilometry systems are also available and provide a similar level of vertical resolution.

### Image processing and measurement protocol

The resulting x, y, and z coordinates were saved in .dat file format and read into the Fiji distribution of ImageJ^47^ using the XYZ2DEM plug-in^48^. This plugin imports the coordinates and interpolates a digital elevation model or DEM using a Delaunay triangulation (Fig. 4D). Scale information (i.e., pixel length, scan area) is available within the SensoSCAN software and can be entered when loading the DEM, and by using the ‘set scale’ function in the ‘analyze’ menu. Once opened, the DEM appears in grey-scale format (Fig. 4E), although shading or shadows can be applied to the DEM to increase contrast, if desired. As the orientation of the DEMs are flipped when they read into ImageJ, we used the transform tab of the menu to flip them vertically before analysis. The line tool was used to draw a transect orthogonal to the feature(s) of interest, across the occlusal shoulders of defects (Fig. 4D) or across several perikymata (Fig. 4B). Due to curvature in both the mesiodistal and cuspo-cervical directions, each transect was drawn across the portion of the DEM that was orthogonal with the microscope during scanning, i.e., level on either side of the defect or perikyma being measured, which is usually in the midline of the DEM.

Once the transect was drawn and a 2D profile plotted, the FindPeaks plug-in was used to locate the Z-coordinate of the peak associated with the occlusal edge of the defect or perikyma ridge, as well as the coordinate for the deepest point within the defect floor or perikyma groove (Figs. 4E, 5C; package can be found within BAR, a collection of Broadly Applicable Routines)^49^. Depending on the length of the transect and the depth of features being targeted, the threshold for the FindPeaks parameters might need modification: for LEH defects, the default settings are usually sufficient (i.e., defects are substantially deeper than any other nearby features like perikymata, and are therefore automatically identified by the software), but particularly when measuring shallow features, the minimum peak amplitude might need to be reduced (e.g., to a value above median local perikymata depth to measure shallow defects, or to a value below median perikymata depth to measure perikymata). The longer the transect drawn within the DEM, the more likely that the default amplitude will need adjustment to identify the grooves. We measured maximum defect depth three times to calculate an average depth for each defect using three separate transects. To measure perikymata depth, the process was the same, except a transect was drawn across just across a few perikymata that occupy a relatively flat area of the crown without obvious defects (Fig. 5B). Given the smaller height of perikymata, and their relatively greater potential for wear over the lifespan, it was particularly important to measure perikymata in unworn to lightly worn teeth. In this study, 10 separate perikymata were measured within different areas of the midcrown in each well-preserved tooth, as evidenced by Tomes’ pit processes within the perikyma grooves^46^.

An alternative approach to collecting defect depths has been proposed^16,21^ in which algorithms are used to eliminate the effects of tooth curvature before extracting 2D measurements from 3D scans. In order to assess the effect of leveling, we used plane and sphere ‘form-removal’ leveling algorithms within SensoSCAN software and compare defect and perikymata depths obtained in raw vs. leveled DEMs. In this study it was not necessary to filter noise before analysis; 2D transects are used to measure the features of interest, making it feasible to avoid the typical aberrations (e.g., bubbles, large scratches, spikes, etc.) when drawing transects. However, if spikes are visible in the DEM and/or resulting 2D profiles, the ‘reduce noise’ function can be used to eliminate outliers before collecting depth data see^17^ for specifications.

### Replicability

To assess the replicability of the imaging and analysis process, we measured the depth of matched defects as well as perikymata depth within the same regions of the midcrown in the same replicas using two models of confocal microscopes hosted at two institutions: the Sensofar S Neox (Université de Bordeaux) and PLu Neox (Universität Tübingen). The mean difference in LEH defect depth between the two microscopes is 2.3% (range = 0.8–3.5%; N = 5), with a mean absolute difference of 1.1 µm (range = 0.3–1.8 µm; N = 5). The mean difference in perikymata depth in the same replicas between the two microscopes is 1.7% (range = 1.1–2.4%; N = 3), with a mean absolute difference of 0.3 µm (range = 0.02–0.77; N = 3).

### Statistical analyses

Assumptions of normality and homogeneity of variance were visually assessed using residual diagnostic plots. Perikymata and defect depth values were natural log-transformed prior to analyses because their distributions are right-skewed. We conducted a pairwise correlation analysis using the Pearson method to assess the relationship between perikymata and defect depth for all teeth in which perikymata were well-preserved (*n* = 29) using the median depths for each tooth. We used separate linear mixed models to test whether there are differences in perikymata and LEH defect depths between the two taxa and between incisors and canines, as well as LEH severity ratios between taxa and among temporal groups (i.e., Neanderthals, Upper Paleolithic, Neolithic, and Medieval). For 12 out of 71 defects, median perikymata depth for the species and tooth type, rather than that exact tooth, was used as perikymata were not well-preserved enough to reliably measure. We included specimen ID as a random effect in all models as multiple defects and perikymata were measured per specimen, and some of the defects are ‘matched’ meaning that they represent the same systemic growth disruption, but on different teeth. Analyses were performed in RStudio (Version 1.3.959)^50^ using package nlme for the mixed models.

## Supplementary Information

Supplementary Information.

## Data Availability

All data analyzed in this study are included in the article and its Supplementary Information files.
