# Supplementary material for: 3D enamel profilometry reveals faster growth but similar stress severity in Neanderthal versus Homo sapiens teeth
Source: Sci Rep. 2021 Jan 12;11:522. doi: 10.1038/s41598-020-80148-w (PMC7804262; doi:10.1038/s41598-020-80148-w)
Supplement: Supplementary file 1 — Supplementary Information. [file 41598_2020_80148_MOESM1_ESM.pdf]

**Supplementary Information**

## 3D enamel profilometry reveals faster growth but similar stress severity in Neanderthal versus *Homo sapiens* teeth

Kate McGrath, Laura Sophia Limmer, Annabelle-Louise Lockey, Debbie Guatelli-Steinberg, Donald J. Reid, Carsten Witzel, Emmy Bocaege, Shannon C. McFarlin, Sireen El Zaatari

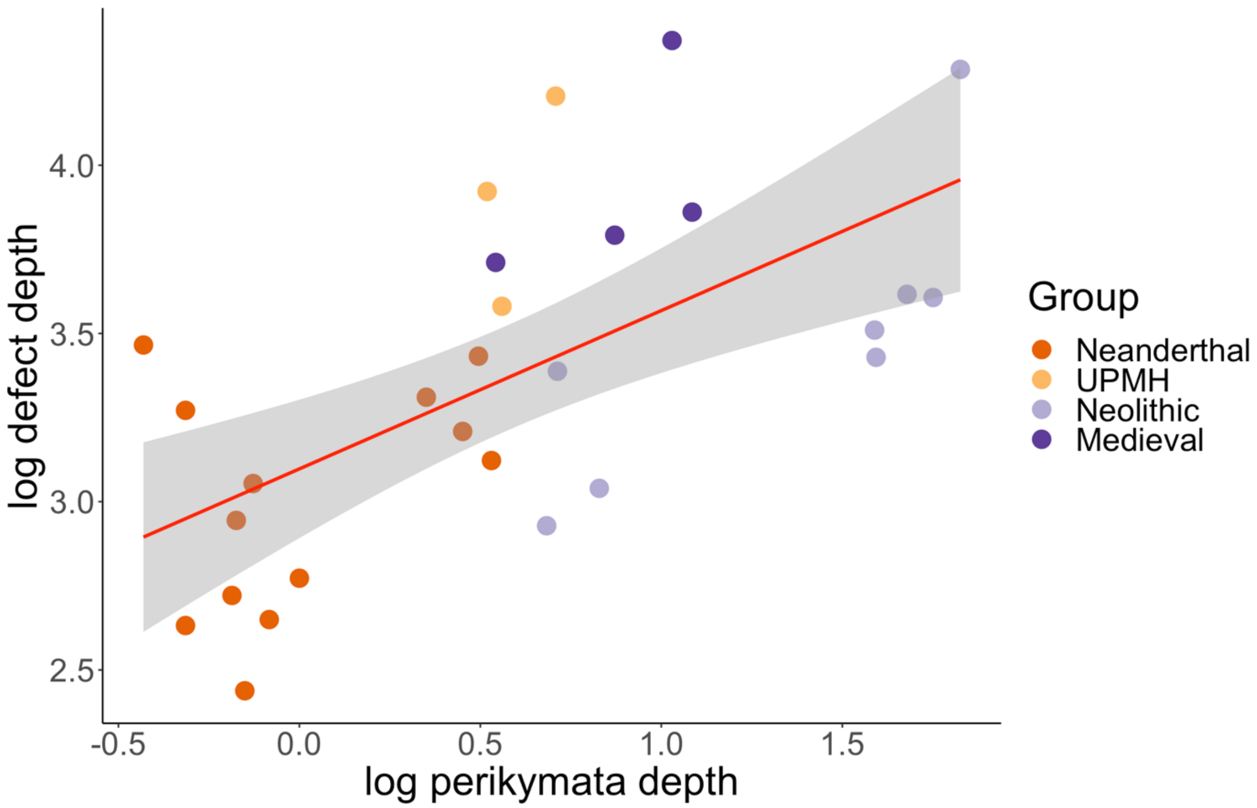


**Figure S1. Regression of log perikymata depth vs. log defect depth.** There is a positive correlation ($R^2$=0.39; p<0.001) between perikymata depth and LEH defect depth in this sample (*n* = 29 replicas). 95% confidence intervals are shown in gray. Figure generated in RStudio (version 1.3.959) [50].

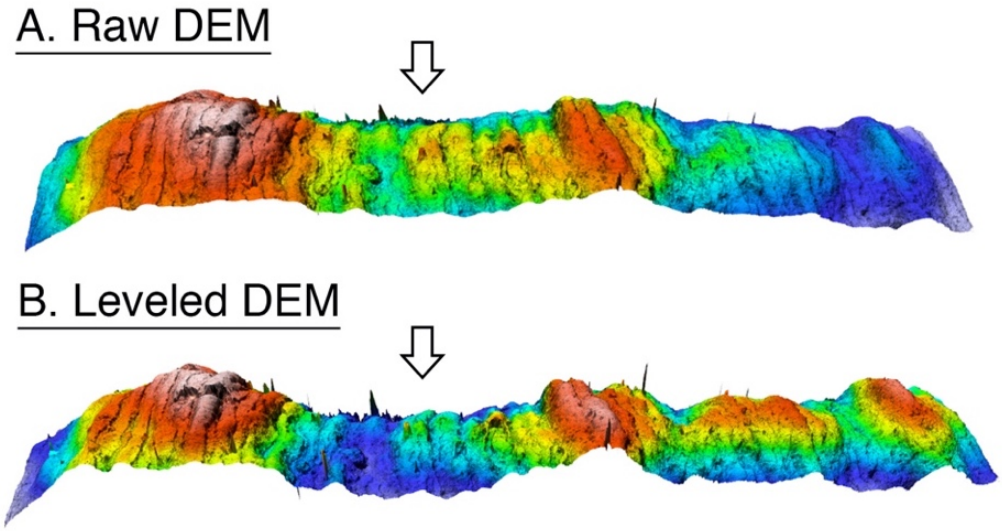


**Figure S2. Raw digital elevation model (DEM) vs. leveled DEM.** Both show a midcrown defect (arrow) and perikymata in the Le Moustier 1 lower canine. **A:** The raw defect, as reported in this study, measures 46.2 μm. **B:** The same defect measured on the leveled DEM measures 35.3 μm. Perikymata show the opposite pattern, with leveled perikymata being 26.8% deeper than raw perikymata in this specimen. Note the exaggerated height of the smaller features like spikes and perikymata in the leveled DEM. The DEMs measure 3.24 x 0.66 mm and were generated within SensoSCAN software (S Neox, https://www.sensofar.com/metrology/products/sneox/).

**Table S1. Perikymata and LEH defect depths by individual**

| Specimen | Tooth type | N pk | Perikymata depth median and range (µm) | N defects | Defect depth median and range (µm) |
|---|---|---|---|---|---|
| Le Moustier 1 – Middle Paleolithic | LRC | 10 | 1.57 (1.17-3.09) | 4 | 24.8 (19.6-46.2) |
| | ULC | 10 | 1.64 (0.77-3.05) | 2 | 31.0 (16.2-45.7) |
| | LLI1 | 10 | 0.83 (0.71-1.18) | 3 | 15.2 (9.8-27.5) |
| | LLI2 | 10 | 0.84 (0.46-1.78) | 2 | 19.0 (10.7-27.3) |
| | ULI1 | - | - | 1 | 18.9 |
| | URI2 | 10 | 0.88 (0.60-1.43) | 4 | 21.9 (16.7-31.3) |
| La Chaise BD10 – Middle Paleolithic | ULI2 | 10 | 0.73 (0.61-0.90) | 2 | 13.9 (10.1-17.7) |
| La Chaise BD11 – Middle Paleolithic | URC | 10 | 0.73 (0.42-1.37) | 2 | 26.4 (24.1-28.6) |
| La Chaise BD12 – Middle Paleolithic | ULI1 | - | - | 1 | 11.1 |
| La Chaise BD15 – Middle Paleolithic | URC | 10 | 0.65 (0.42-1.08) | 1 | 32.0 |
| La Chaise BD16 – Middle Paleolithic | URC | 10 | 1.00 (0.80-1.94) | 3 | 16.0 (13.3-21.7) |
| La Chaise Suard S41 – Middle Paleolithic | ULI1 | 10 | 1.70 (0.90-3.05) | 1 | 22.7 |
| Biache-Saint-Vaast 1 – Middle Paleolithic | ULI1 | 10 | 0.92 (0.53-1.27) | 2 | 14.2 (12.9-15.4) |
| Kulna 1 A17 092 – Middle Paleolithic | URC | 10 | 1.42 (1.05-2.14) | 2 | 27.4 (16.3-38.5) |
| Monsempron 3 – Middle Paleolithic | URC | - | - | 3 | 28.6 (18.2-31.6) |
| Monsempron 4 – Middle Paleolithic | URI1 | 10 | 0.86 (0.49-1.45) | 2 | 11.5 (9.5-13.4) |
| Rochelot 1 – Middle Paleolithic | LLC | - | - | 1 | 30.3 |

| | | | | | |
|---|---|---|---|---|---|
| Les Rois Mandible B – Upper Paleolithic | LRC | 10 | 2.03 (1.28-4.43) | 2 | 67.1 (52.9-81.2) |
| | LRI1 | 10 | 1.75 (0.96-2.45) | 2 | 35.9 (33.6-38.2) |
| | LRI2 | 10 | 1.68 (0.83-2.72) | 1 | 50.5 |
| Saint Germain-la-Rivière 1970-8-B4 – Upper Paleolithic | LLI1 | - | - | 2 | 30.1 (21.5-38.6) |
| | LLI2 | - | - | 3 | 39.0 (34.0-43.9) |
| Çatalhöyük 1938.1 – Neolithic | LRC | 10 | 6.21 (4.45-11.29) | 2 | 72.6 (43.5-101.7) |
| | LRI1 | 10 | 4.92 (3.85-7.95) | 2 | 30.9 (20.6-41.1) |
| | LRI2 | 10 | 4.90 (2.83-8.17) | 2 | 33.5 (27.5-39.4) |
| Çatalhöyük 1913.1 – Neolithic | LLC | 10 | 5.76 (3.91-7.39) | 2 | 36.9 (32.8-40.9) |
| | ULC | 10 | 5.40 (3.64-8.47) | 2 | 37.2 (32.7-41.7) |
| | LRI2 | 10 | 2.29 (1.58-3.27) | 1 | 20.9 |
| | URI1 | 10 | 1.98 (1.22-3.09) | 1 | 18.7 |
| | ULI2 | 10 | 2.04 (1.55-3.42) | 1 | 29.6 |
| Saxon burial at Hildesheim Befund 1701 – Medieval | LLC | 10 | 2.96 (1.47-4.21) | 5 | 47.5 (38.8-84.8) |
| | URI1 | 10 | 1.72 (1.14-2.24) | 3 | 40.9 (37.2-52.4) |
| Saxon burial at Hildesheim Befund 953 – Medieval | ULC | 10 | 2.80 (1.68-5.30) | 1 | 79.1 |
| | LLI1 | 10 | 2.39 (1.24-2.54) | 2 | 44.4 (40.3-48.4) |
| | URI1 | - | - | 2 | 19.2 (18.8-19.5) |

**Table S2. LEH defect depths of the same disruptions matched across the anterior teeth**

| Specimen | Defect name | Matched defect depth |
|---|---|---|
| Le Moustier 1 | defect 1 | LRC – 46.2<br>ULC – 45.7<br>LLI1 – 27.5<br>LLI2 – 27.3<br>ULI1 – 18.9<br>URI2 – 24.0 |
| Les Rois Mandible B | defect 1 | LRC – 81.2<br>LRI1 – 33.6<br>LRI2 – 50.5 |
| Saint Germain-la-Rivière 1970-8-B4 | defect 1 | LLI1 – 38.6<br>LLI2 – 43.9 |
| | defect 2 | LLI1 – 21.5<br>LLI2 – 34.0 |
| Çatalhöyük 1938.1 | defect 1 | LRC – 101.7<br>LRI2 – 39.4 |
| Çatalhöyük 1913.1 | defect 1 | LLC – 40.9<br>ULC – 41.7<br>URI1 – 18.7<br>ULI2 – 29.6 |
| | defect 2 | LLC – 32.8 |

| | | |
|---|---|---|
| | | ULC – 32.7<br><br>LRI2 – 20.9 |
| Saxon burial at Hildesheim Befund 1701 | defect 1<br><br><br><br><br>defect 2 | LLC – 58.8<br><br>URI1 – 37.2<br><br><br>LLC – 84.8<br><br>URI1 – 40.9 |

N.B. This table is not a complete account of all LEH defects present on these teeth or the sample, rather those that could be reliably matched on at least two teeth belonging to the same individual.
